# Genetic Markers in Biological Fluids for Aging-Related Major Neurocognitive Disorder

**DOI:** 10.2174/1567205012666150302155138

**Published:** 2015-03

**Authors:** S.A. Castro-Chavira, T. Fernández, H. Nicolini, S. Diaz-Cintra, R.A. Prado-Alcalá

**Affiliations:** 1 Departamento de Neurobiología Conductual y Cognitiva, Instituto de Neurobiología. Campus UNAM Juriquilla, Universidad Nacional Autónoma de México. C. P. 76230. Querétaro, México;; 2 Laboratorio de Genómica de Enfermedades Psiquiátricas y Neurodegenerativas. Instituto Nacional de Medicina Genómica. C. P. 14610. Distrito Federal, México;; 3 Departamento de Neurobiología del Desarrollo y Neurofisiología, Instituto de Neurobiología. Campus UNAM Juriquilla, Universidad Nacional Autónoma d e México. C. P. 76230. Querétaro, México.

**Keywords:** Alzheimer´s disease, cognitive impairment, dementia, frontotemporal lobar degeneration, genetic markers, Lewy body disease, neurocognitive disorder, Parkinson´s disease, vascular disease

## Abstract

Aging-related major neurocognitive disorder (NCD), formerly named dementia, comprises of the different acquired diseases whose primary deficit is impairment in cognitive functions such as complex attention, executive function, learning and memory, language, perceptual/motor skills, and social cognition, and that are related to specific brain regions and/or networks. According to its etiology, the most common subtypes of major NCDs are due to Alzheimer’s disease (AD), vascular disease (VaD), Lewy body disease (LBD), and frontotemporal lobar degeneration (FTLD). These pathologies are frequently present in mixed forms, i.e., AD plus VaD or AD plus LBD, thus diagnosed as due to multiple etiologies. In this paper, the definitions, criteria, pathologies, subtypes and genetic markers for the most common age-related major NCD subtypes are summarized. The current diagnostic criteria consider cognitive decline leading to major NCD or dementia as a progressive degenerative process with an underlying neuropathology that begins before the manifestation of symptoms. Biomarkers associated with this asymptomatic phase are being developed as accurate risk factor and biomarker assessments are fundamental to provide timely treatment since no treatments to prevent or cure NCD yet exist. Biological fluid assessment represents a safer, cheaper and less invasive method compared to contrast imaging studies to predict NCD appearance. Genetic factors particularly have a key role not only in predicting development of the disease but also the age of onset as well as the presentation of comorbidities that may contribute to the disease pathology and trigger synergistic mechanisms which may, in turn, accelerate the neurodegenerative process and its resultant behavioral and functional disorders.

## INTRODUCTION

The risk for suffering dementia represents a growing concern as human life expectancy increases around the world in recent years. Dementia is a multicausal disease that the World Health Organization (WHO) in its International Classification of Diseases (ICD-10) defines as “a syndrome due to disease of the brain - usually of a chronic or progressive nature - in which there is disturbance of multiple higher cortical functions, including memory, thinking, orientation, comprehension, calculation, learning capacity, language, and judgment… The impairments of cognitive function are commonly accompanied, and occasionally preceded, by deterioration in emotional control, social behavior, or motivation” [1].

According to The National Institute of Aging-Alzheimer's Association (NIA-AA), diagnosis of dementiarequires cognitive or behavioral symptoms in two of the five following domains: memory, executive function, visuospatial performance, language, and personality/behavior [2]. The recently published DSM-5 includes a chapter entitled “Neurocognitive Disorders” instead of the previous “Delirium, Dementia, and Amnestic and Other Cognitive Disorders” chapter in DSM-IV. DSM-5 considers that the term “dementia” is most often used for aging-related diseases such as Alzheimer's and Lewy Body; experts in cerebrovascular disease and frontotemporal lobar degeneration prefer other more specific terms; and cognitive loss caused by traumatic brain injury or HIV infection in the younger is not typically named dementia. As a result, DSM-5 renames dementia as ‘major neurocognitive disorder (NCD)’ and mild cognitive impairment (MCI) as ‘mild NCD’. NCDs include a variety of ages and etiologies, and are defined as acquired disorders in which the primary clinical deficit is present in at least one cognitive function. These cognitive functions are associated to particular brain regions, neural pathways, or cortical/subcortical networks [3], and they include complex attention, learning and memory, executive ability, language, visuoconstructional-perceptual ability, and social cognition [4].

In this paper, the terms “major NCD” and “dementia” are used indistinctly and comprise the different aging-related diseases whose primary deficit is impairment in cognitive functions related to particular brain regions and/or networks. The most common aging-related subtypes of major NCD according to their etiologies are due to Alzheimer’s disease (AD), vascular disease (VaD), Lewy body disease (LBD), frontotemporal lobar degeneration (FTLD), Parkinson´s disease (PD), and multiple etiologies [1], with AD accounting for 23.6-51% of major NCD cases [5]. However, *post mortem* studies suggest that many people with dementia have mixed AD and VaD or AD and LBD pathologies. These ‘mixed dementias’ are underdiagnosed [6] and very prevalent; e.g., in one series of 1050 post-mortem studies in elderly demented individuals, while 86% had AD-related pathology, only 43% had pure AD [7].

Differences in pathology progression between dementia types remain unclear. Patients diagnosed with primary clinical VaD showed slower neuropsychological decline when compared with those diagnosed with AD after adjustment for age, sex, race, marital status, education, comorbidities, years since symptom onset, and cognitive status in a study of functional decline comparing AD, VaD, and LBD; however, there were no significant differences between LBD and AD or VaD [8]. A more recent study comparing the progression of cognitive decline in LBD, AD, and the mixed pathologies (LBD plus AD) did not report significant differences in the speed of the overall cognitive decline progression but did find differences in the progression of the decline of specific cognitive processes. However, in the mixed LBD and AD NCD there were more cognitive processes in which the decline was faster than in each of these separated dementias [9].

Another aspect recently considered by the International Working Group (IWG) and NIA-AA is the recognition that the underlying neuropathology of the disease begins before the manifestation of symptoms, thus they included in their classifications an asymptomatic or presymptomatic phase for which biomarkers now exist [10]. 

## DEMENTIA SUBTYPES AND THEIR BIOMARKERS

### Alzheimer´s Disease (AD)

AD is defined by NIA-AA as cognitive impairment that interferes with work or daily activities, represents a decline from a previous level, and cannot be explained by other disorders, including psychiatric disorders [10]. The pathological features that characterize AD are neuronal atrophy, synapse loss, abnormal accumulation of amyloid-β protein (Aβ) forming senile plaques, and hyperphosphorylated tau protein forming neurofibrillary tangles (NFTs) [11]; see Fig. (**1**).

Aβ is a 38-43 amino acid fragment of the amyloid precursor protein (APP) cleaved by β-secretase and *γ*-secretase. Amino acids 1-28 of Aβ are extracted from the APP region out of the plasma membrane, and amino acids 29-43 of Aβ are extracted from the membrane-spanning region. The APP intracellular fragment seems to have a transcriptional function. Whether Aβ has a function itself in the brain remains to be clarified [12]. Aβ deposits in the form of plaques beginning in parietal, temporal, and frontal association areas. Degeneration in basal forebrain structures (*nucleus basalis*, diagonal band of Broca, *substantia innominata*, and medial septal nuclei) results in a major reduction of cholinergic projections to the cortex, and limbic structures, including the hippocampus [11]. APP has essential physiological functions in synaptic processes, including transcellular synaptic adhesion [13], and is an essential copper-binding protein altering metal homeostasis [14,  15]. As well, copper levels modulate APP cis- and trans-cellular dimerization, which in turn regulates the physiological function of APP and affects its pathogenicity in AD [16]. In addition, in Down syndrome, AD is assumed to be caused by the triplication and overexpression of the APP gene, located on chromosome 21. High plasma concentrations of Aβ1-40 and Aβ1-42 are determinants of the risk of NCD in persons with Down syndrome [17]. Aβ1-40 (Aβ40) is normally the most abundant Aβ species. Aβ1-42 (Aβ42) is more hydrophobic and prone to aggregate than Aβ40 and, thus, seems to have a key role initiating Aβ aggregation [12]. Studies about the relationship between plasma/serum Aβ and AD reported no difference in plasma/serum Aβ levels (Aβ40 and Aβ42) between sporadic (late-onset) AD and controls. In contrast, plasma Aβ42 was found to be increased and Aβ40 decreased in individuals with familial AD (early-onset) [18]. Mutations in presenilin 1 (PS-1), the catalytical subunit of the protease γ-secretase, result in familial AD. PS-1 familial AD-linked mutations associated with decreased γ-secretase activity result in altered membrane integration and catalytic site conformation, mechanisms associated to increased Aβ42/Aβ40 ratio [19]. Longitudinal studies indicated that Aβ42 levels in plasma were significantly higher in cognitively normal individuals who converted to AD as compared to non-converters. Over time, Aβ42 levels decreased in converters to AD, suggesting that plasma Aβ42 might be a marker for progression, and not a diagnostic marker [18]. 

Plasma quantifications of Aβ40 and Aβ42 have not shown correlation either with brain Aβ, plaque levels [18] or CSF Aβ42 or Aβ40 [20]. The presence of Aβ deposition in the brain can be detected by amyloid imaging in combination with the measurement of CSF Aβ42 concentration. Before AD clinical onset, some NFT development is present, and the acceleration of tau aggregation and neurodegeneration may mark the transition to the symptomatic phase. Increases in the CSF of total and phosphorylated tau begin 3-4 years before the onset of mild NCD, and make this transition detectable. The earliest symptoms and signs of cognitive decline caused by AD appear when Aβ accumulation in the brain is already approaching its maximal extent. On the other hand, moderate to severe dementia is accompanied by a peak in NFTs, oxidative stress, inflammation, synaptic and network dysfunction, and neuronal cell death. By the time mild NCD is present, densities of plaques and tangles are substantial, and neuronal loss is also significant in certain brain regions [12]. The CSF tau increase observed in AD may be explained by the release of tau from degenerating neurons and its subsequent diffusion [20].

Abnormal tau hyperphosphorylation is produced by a phosphorylation/dephosphorylation imbalance in the AD brain apparently caused by decreased protein phosphatase-2A activity. Brain tau is approximately three- to four-fold more hyperphosphorylated in AD than the normal. On one hand, AD phosphorylated tau sequesters normal MAPs from microtubules causing microtubule inhibition and disruption [21]. This microtubule disruption, in turn, possibly leads to axon degeneration (as Mandelkow and Mandelkow [22] propose), increasing tau levels in the cytosol [20]. On the other hand, AD phosphorylated tau bound to normal tau forms oligomers. This tau oligomers are sedimentable and self-assemble into paired helical and straight filaments in the form of NFTs, unlike the highly-soluble normal tau [21]. Tangle-intrinsic tau changes from full-length isoforms to microtubule binding region-only fragments, suggesting a significant role of proteolysis at both tau C and N termini in NFT formation. Truncated C-terminal tau fragments can sequester full-length tau in both mutant and wild-type forms [23]. NFTs are formed first in medial temporal structures (e.g., hippocampus and entorhinal cortex) and, as the disease progresses, extend to association areas in temporal, parietal, and frontal cortices [11]. These structures are, as above mentioned, also susceptible to amyloid-plaque damage, thus they frequently exhibit a combined pathology. In a study that compared different predictors using the Alzheimer´s Disease Neuroimaging Initiative data, researchers found that fluorodeoxyglucose-positron emission tomography (FDG PET) and episodic memory performance were the strongest predictors of progression from MCI to AD, whereas CSF tau and Aβ combined with FDG-PET were predictive of cognitive decline onset [24].

The main risk factor for most forms of dementia is advanced age. Onset before 65 years of age (early-onset AD) is very unusual and often suggests single gene mutations in at least one of three loci: APP, PS-1, and PS-2 [6]. The gene for APP maps on chromosome 21q21.2, and at least 7 different mutations in this gene cause early-onset familial AD. They are all missense mutations that change α-, β-, or γ-secretase cleavage sites, altering the normal APP proteolysis. The PS-1 gene is located on chromosome 14q24.3, and mutations in this gene account for most of autosomal familial AD cases. PS-1 codes for an integral membrane protein with 467-amino acids and eight transmembrane domains. PS-1 has been proposed to be involved in protein and membrane trafficking and in the regulation of intercellular signal transduction. Early-onset familial AD has been associated with more than 60 mutations of this gene, being most of them missense mutations. The PS-2 gene is located on chromosome 1q31-q42 and codes for a 448-amino acid protein which shares 67% of homology with the PS-1protein, implying similar functions. Early-onset familial AD has been associated with two missense mutations of the PS-2 gene. Indirect evidence suggests that PS-1 and PS-2 proteins may mimic or cooperate with γ-secretase in APP processing [25]. Studies in individuals with autosomal dominant mutations leading to the development of early-onset AD show signs of disease long before onset: amyloid accumulation beginning at 20 years, neurodegeneration biomarkers at 10 years, and neuropsychological (Logical Memory Recall test) deficits 5 years before onset [26].

For late-onset AD, apolipoprotein E (ApoE) ε4 allele is the strongest genetic risk factor currently known, conferring an approximate 3-fold increased risk in those who carry one copy of this allele compared with non-carriers [12]. Human ApoE is a 35 kDa glycoprotein that exits in three isoforms (ε2, ε3, and ε4) which differ in sequence by one amino acid [27]. ApoE is an important regulator of lipoprotein metabolism in plasma, and plays a role in diverse processes including cholesterol transport, neuronal plasticity, and inflammation in the brain. Evidence suggests that ApoE influence the clearance of soluble Aβ and the aggregation propensity of Aβ by acting as an Aβ binding molecule. Approximately 50% of patients with AD are ε4-positive, and Aβ deposition occurs earlier and to a greater extent in this population compared to ε4-negative patients. Interestingly, two copies of ε4 result significantly more pathological than one [12]. Age, family history, and head injury with loss of consciousness are epidemiological risk factors that affect AD risk. Furthermore, the risk that head injury leads to AD is higher (OR = 15-20) in patients with at least one ApoE ε4 allele. On the other hand, higher education levels are associated with lower AD risk, may be due to the increase of cognitive reserve associated to more years of instruction [12].

The evaluation of single nucleotide polymorphisms (SNPs) through genome-wide association studies (GWAS) has provided evidence for new genetic risk factors, e.g. mutations in genes encoding apolipoprotein J/clusterin (CLU), phosphatidylinositol-binding clathrin assembly protein (PICALM), complement receptor 1 (CR1) [10, 18-21], bridging integrator protein 1 (BIN1) [12, 28, 30, 31], disabled homolog 1 (DAB1) [18], sialic acid binding Ig-like lectin (CD33), membrane spanning 4A gene cluster (MS4A), CD2-associated protein (CD2AP), Ephrin receptor A1 (EPHA1) [12,  30,  31], translocase of outer mitochondrial membrane 40 homolog (TOMM40) [12], ATP-binding cassette transporter (ABCA7) [12,  30], FERM domain containing 4A (FRMD4A) [30], major histocompatibility complex class II DRβ5 and DRβ1 (HLA-DRB5, HLA-DRB1), protein tyrosine kinase 2β (PTK2B), sortilin-related receptor L(DLR class) 1 (SORL1), and solute carrier family 24 (sodium/potassium/calcium exchanger) member 4 (SLC24A4) - Ras and Rab interactor 3 (RIN3) [31].

CLU is the most likely candidate for a new AD-associated gene. CLU protein seems to regulate the toxicity and conversion of Aβ into insoluble forms. CLU levels are elevated in certain conditions involving brain injury or chronic inflammation. In AD patients, the CLU protein has been found in amyloid plaques and the CSF. Moreover, CLU expression is increased in the cortex areas presenting the pathology. Also, APOE and CLU proteins cooperate in suppressing Aβ deposition. CLU levels increase according to the number of APOE-ε4 alleles, suggesting an induction of CLU in individuals with low protein levels of ApoE [28].

PICALM, another gene consistently associated with AD, is prominently expressed at pre- and postsynaptic sites in neurons. PICALM is associated to clathrin-mediated endocytosis, and appears to direct the protein trafficking prominent in the fusion of synaptic vesicles to the presynaptic membrane during neurotransmitter release. Stereological and biochemical analyses have shown that cognitive defects correlate better with the AD-related reduction in synaptic density than with the accumulation of plaques and tangles. PICALM mutations could affect the risk of AD through endocytotic processing of APP, increasing Aβ production and release as a result. When the synaptic activity increases, more APP is taken into endocytotic compartments to be cleaved [28].

CR1, ABCA7, CD33 and EPHA1 are involved in the function of the immune system; BIN1, CD33, CD2AP participate in endocytosis and other processes of the cell membrane, and ABCA7 participates also in the lipid metabolism [12]. Surprisingly, a case control study with two Canadian populations found that (beyond age, sex, level of education, and ApoE ε4 status) only SNPs in CR1, TOMM40, BIN1, and CD33 were significant risk factors for late-onset AD [32]. The population-attributable fractions for the above mentioned candidate genes are between 2.72-5.97%, compared to 20% for ApoE ε4. There are SNPs (only seen in people with Aβ accumulation) in the MAPT gene and in protein phosphatase B (calcineurin), that do not affect AD progression. Evidence suggests that several cellular proteins, including RAGE, Fyn, tau, NADPH oxidase, prion protein, and EphB2 among others, may be mediating the damaging effects of Aβ and tau on neurons, glia, and the cells forming the neurovascular unit [12].

### Vascular Disease (VaD)

Vascular cognitive disorder (VCD) is caused by ischemic and/or vascular lesions in different brain areas and neuronal networks and may result in deafferentation of frontal and limbic cortical structures and interruption of basal ganglia-cortical, cortico-cortical, and ascending pathways. Dementia related to vascular disorders is now described as “vascular dementia” (VaD) and “vascular cognitive impairment” (VCI). Some models explain the resultant cognitive decline in terms of damaged cortical and subcortical circuits and interactions with frontal-subcortical lesions or frontal atrophy [33]. Clinical data revealed an incidence of 15.8%, and a prevalence of VaD alone of 0.3% in 65-69 year-olds, which increased to 5.2% in individuals after 90 [34].

The National Institute of Neurological Disorders and Stroke and L'Association Internationale pour la Recherche et l’Enseignement en Neurosciences (NINDS-AIREN) criteria used in VaD clinical trials require neuroimaging evidence of focal brain damage, focal clinical signs, and cognitive deficits linked to functional impairment in at least three cognitive domains, one of which must be memory [35]. Clinico-pathologic studies reported moderate sensitivity (average 50-56%) and variable specificity (range 64-98%, average 87%) for these criteria. The vessel disorders most frequently associated with VaD are atherosclerosis of cerebral arteries (AS), arteriosclerosis or cerebral small vessel disease (SVD), and cerebral amyloid angiopathy (CAA) [33]. VCI/VaD is the consequent pathology from vascular lesions producing cognitive deterioration. Patients with VCI exhibit multifold pathological changes which include a variety of large and small cerebrovascular and ischemic lesions of different extensions: focal, multifocal, or diffuse. There are three different patterns of vascular brain lesions that produce cognitive deterioration: 1. multi infarct VaD, 2. strategic infarct VaD, and 3. subcortical vascular encephalopathy. In multi infarct VaD, multiple micro, lacunar, and small or large infarcts are distributed throughout the gray matter. Strategic infarct VaD is characterized by infarcts in regions which play a key role in cognitive function. Subcortical vascular encephalopathy involves lesions in central and peripheral white matter that may be accompanied by small infarcts [36].

C-reactive protein, lipids, homocysteine, glucose, hemoglobin A1C, insulin, clotting factors, and fibrinogen have been proposed as plasma markers for VaD, even though CSF analyses have been more successful than those of blood to distinguish between different cognitive pathologies. VCI and subcortical ischemic vascular dementia (SIVD) patients show increased amounts of CSF albumin indicating a compromised BBB. A greatly increased CSF concentration of the neurofilament light subunit in SIVD patients is associated with white matter changes, which are consistent with the axonal damage characteristic of VCI [37].

VCI is present in several monogenic disorders (associated genes in parentheses): cerebral autosomal dominant arteriopathy with subcortical infarcts and leukoencephalopathy, CADASIL (NOTCH 3); hereditary variants of CAA (APP, CYSTATIN C, and other genes); sickle-cell disease (HBB and other hemoglobin genes); Fabry disease (GLA); homocystinuria (CBS and other genes) [37]. It has been proposed that genetic risk associated to VaD consist of predisposing genes and genes regulating brain response to cardiovascular insult [38]. The two best studied monogenic forms of cerebrovascular disease are CADASIL and hereditary cerebral hemorrhage with amyloidosis-Dutch type (HCHWA-D) [39]. CADASIL is the most prominent inherited form of SVD in relatively young adults [35, 40]. The main clinical features include migraine with aura, subcortical ischemic events, mood disturbances, and cognitive decline. The disease, usually manifesting before 60 years of age, has a progressive course, with severe disability and dementia in the advanced stage. The pathologic hallmark of the disease is the presence of granular osmiophilic material on electron microscope examination of the smooth muscle cells in cerebral and extracerebral vessels, including dermal arterioles [40]. CADASIL results from mutations in the Notch3 gene, which participates in the regulation of smooth muscle cell proliferation and differentiation [39]. Bianchi *et al.* [40] recently reported a Notch3 deletion mutation in intron 3 that leads to aberrant splicing and is associated with a pathologically confirmed CADASIL phenotype.

HCHWA-D is a syndrome of hemorrhagic strokes and cognitive impairment characterized by abnormal amyloid deposition in the walls of leptomeningeal arteries and cortical arterioles due to a mutation in the APP gene [39]. Furthermore, the Aβ E22Q mutation of HCHWA-D is related to VaD presence [41].

A bead-based proteome study analyzing sera of 30 VCI patients and 30 healthy controls identified twenty-four peptides whose expression differed significantly between groups. The peptide peak chosen for analysis matched the amino acid sequence of a fragment of the trace amine-associated receptor 6 (TAAR6); the sensitivity of the model was 95% and 80%, internally and externally validated, respectively, and whose specificity was 100% for both [42]. In a recent study, Kim *et al.* [43] suggested an association of VaD risk with a sequence variant of rs290227 in the spleen tyrosine kinase (SYK) gene that causes intron retention which, in turn, renders mature transcripts inappropriately. Another study in this respect, associated the ApoE-ε4 allele with infarct/stroke dementia and SIVD, and the MTHFR gene with SIVD alone [44].

**Fig. (2):**
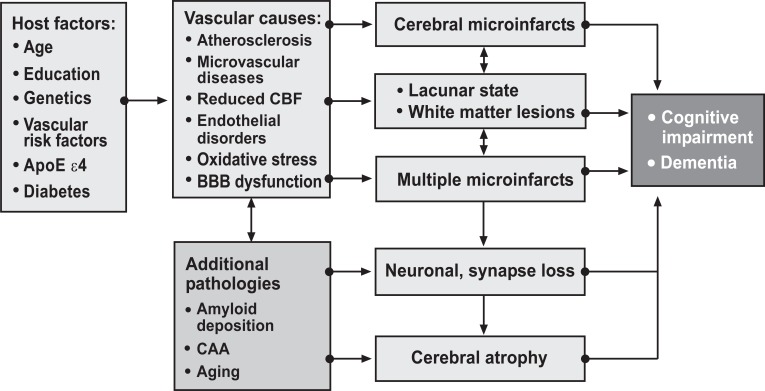
Pathogenic factors for VCI/VaD. Modified from [33].

VCI has been associated with hypertension, diabetes, dyslipidemia, tobacco smoking, atrial fibrillation (Fig. **2**), and novel risk factors, e.g. metabolic syndrome [45].

### NCD due to Multiple Etiologies (Vascular Disease Plus Alzheimer´s Disease)

Senile mixed dementia (MD) was first described as the association of vascular and degenerative lesions in the same demented patient by Delay and coworkers in 1962. 

Recent studies indicate that there is a high percentage of cases presenting combined AD and cerebrovascular disease pathologies which may affect cognitive function through additive and synergistic interplays. AD and VaD are the most prevalent NCDs in the elderly; however, the distinction between isolated AD, VaD, and MD, remains one of the most difficult diagnostic challenges [46]. The incidence and prevalence of MD as evidenced by the combination of autopsy-proven Aβ plaques, NFTs, and multiple vascular or ischemic lesions is unknown, e.g. diverse autopsy studies provide prevalence estimates between 2 and 58%. A candidate differential diagnostic feature is that in AD and VCI, the vascular lesions frequently involve subcortical regions or are multiple microinfarcts, meanwhile in MD, large/hemispheral infarcts and multiple microinfarcts are more frequent. This features indicate also different mechanisms for each pathology [47]. A phenotypic analysis reported four different types of MD: mild/mixed dementia in 40.80%, moderate/mixed dementia in 24.21% of cases, dysexecutive in 21.97%, and amnestic in 13% [46,  48].

A study exploring genetic variants within the ApoE gene transcriptional regulatory region in association with sporadic AD, VaD and MD did not find any interaction for MD [49]. A lipidomics analysis reported that the levels of sulfatides and lysobisphosphatidic acids were progressively increased from control nondemented to SIVD to MD subjects in the temporal cortex gray matter. White matter phospholipid profiles indicated elevated membrane degradation in MD [50]. Kim and coworkers [51] observed no susceptibility associations to VaD when 13 SNPs previously associated with AD were analyzed in a study with 207 VaD patients and 207 sex- and age-matched controls. Also, decreased linkage to 20p13 including the ANGPT4 gene in families with MD was also reported [52]. As a result, clear genetic markers for this NCD due to multiple etiologies remain to be found.

### Lewy Body Disease (LBD)

Lewy Body Disease (LBD) is a neurodegenerative disorder that affects cognition, behavior, movement, and autonomic function characterized by fluctuating cognition, spontaneous Parkinsonism, and recurrent visual hallucinations [53]. It is estimated that LBD occur in 0% to 5% of individuals and that it accounts for 0% to 30.5% of NCD cases. According to the LBD Consortium, a diagnosis of LBD can be made when at least two of the three above-mentioned core features, or one core feature and one suggestive feature (repeated falls, syncope, transient loss of consciousness, neuroleptic sensitivity, systematized delusions, non-visual hallucinations) are present [54,  55].

LBD features a burden of α-synuclein (αSyn) pathology with widespread cortical Lewy bodies and AD-related pathology [56]. Synucleins are 123-to-143-amino-acid proteins localized in presynaptic terminals which may be involved in neurotransmission and/or synaptic organization. Lewy bodies are spherical structures found in the cytoplasm [54] of brainstem cells, specifically in the *substantia nigra* and *locus coeruleus*; however, these structures can also be found in the amygdala, frontal, cingulate, and inferior temporal cortices, and the peripheral nervous system [55].

Rapid eye movement sleep behavior disorder has been proposed to represent the prodromal phase of LBD and Parkinson´s disease, as shown in a study with 44 participants, 36 of whom (82%) had developed a defined neurodegenerative syndrome after a 9- to 14-year follow-up, and patients who remained disease-free showed decreased striatal DAT binding [57].

Currently, there are no blood or CSF markers which can be used for diagnosis, to follow disease progression, or as an outcome parameter for intervention in LBD [53]. Among the studies aiming to set LBD genetic markers, Nalls *et al.* [58] found a significant association between glucocerebrosidase (GBA1) mutation carrier status and LBD, and the GBA1 association with Parkinson’s disease was confirmed. Also, a study with 150 AD patients, 50 LBD patients, and 279 healthy elderly controls showed that annexin A5 and ApoE ε4 are common plasma markers for AD and LBD [59]. Yet, Mulugeta *et al.* suggested CSF amyloid-38 as a diagnostic biomarker for LBD using the A-42/A-38 ratio to distinguish AD from LBD (with 78% sensitivity and 67% specificity) [53]. Moreover, missense mutations of the αSyn gene in exons 3 and 4 have been reported in familial LBD cases, but these findings could not be replicated by other studies. In addition, familial LBD has been strongly associated to a region of chromosome 2, 2q35-q36 [54,  60].

### Parkinson´s Disease

In NCD due to Parkinson's disease, the motor and other symptoms of Parkinson's disease are present at least one year before major NCD is established, whereas symptoms begin shortly before, or concurrent with, motor symptoms in major or mild NCD. Approximately 75% of the individuals with Parkinson's disease develop major NCD [5]. There is a synergistic pathological effect derived from the interaction between αSyn and AD pathology that is responsible for the cognitive decline in NCD due to Parkinson´s disease. A number of genetic factors is associated to changes in the risk for presenting NCD due to Parkinson´s disease, i.e. triplications in the αSyn gene increase this risk, while parkin mutations reduce this risk [61]. A recent study with 343 Parkinson´s disease patients, out of which 72 patients presented NCD, reported that serum uric acid levels were not different between Parkinson´s disease patients with or without major NCD and no significant association was detected between any single SNP and the risk of NCD due to Parkinson´s disease [62].

### Frontotemporal Lobar Degeneration (FTLD)

Frontotemporal lobar degeneration (FTLD) comprises a group of diverse neurodegenerative disorders of unknown etiology that may lead to early-onset major NCD and can appear before 30 years-old as well as during aging. Most FTLD cases are diagnosed between 45 and 64 years of age [63, 64]. According to the frontotemporal dementia consensus criteria, FTLD encompasses progressive degenerative changes in behavior, executive function, and/or language, which can be separated into three syndromes: behavioral frontotemporal dementia, semantic dementia, and progressive non-fluent aphasia. The behavioral variant includes progressive behavioral and executive function decline with predominantly-frontal atrophy. Semantic dementia comprises anomia and asymmetrical anterior temporal atrophy. Progressive non-fluent aphasia comprehends motor speech deficits with predominantly left peri-sylvian atrophy [64]. FTLD pathology includes striatum degeneration and bilateral atrophy of the frontal and anterior temporal lobes [63]. Currently utilized CSF biomarkers for the evaluation of neurodegenerative diseases include total tau protein (t-tau), phosphorylated tau 181 (p-tau 181), and Aβ42. A recent study found FTLD to be associated with a higher Aβ42:Aβ40 ratio compared to AD [64].

Excluding depression, one-third to one-half of familial FTLD cases present an autosomal dominant inheritance pattern and up to 40% of total FTLD cases report a family history of neurodegenerative illness [65]. Autosomal dominant FTLD has been importantly associated to specific genes, i.e. (i) MAPT, encoding the microtubule-associated protein tau, (ii) PGRN, encoding the protein progranulin, and (iii) C9ORF72, a hexanucleotide repeat expansion on chromosome 9. Four other genes are responsible for a minority of FTLD cases as follows: VCP (vasolin-containing protein), CHMP2B (chromatin-modifying protein 2B), TDP-43 (transactive DNA-binding protein), and FUS. As well, mutations in TARDBP have been described. Currently, no *in vivo* biomarkers have been identified that reliably reflect the underlying neuropathology of FTLD, such as tau, TDP-43, or FUS [64]. In 2005, mutations in the gene coding for CHMP2B, located on chromosome 3p11.2, were discovered in a large Danish cohort with familial FTLD [66].

Tau mutations on chromosome 17, which cause autosomal dominant frontotemporal dementia with Parkinsonism (FTDP-17), increase 4-R:3-R tau ratio or missense protein mutations, both of which are more easily abnormally hyperphosphorylated than the wild-type protein. The missense tau mutations G272V, P301L, V337M, and R406W are more readily hyperphosphorylated and self-aggregated into filaments, which may be responsible for the early onset, severity, and autosomal dominance in FTDP-17 [21].

## DISCUSSION

As a nosologic entity, dementia constitutes a wide group of diseases with common behavioral, pathologic, and genetic characteristics. Over the years, the description of newly discovered genes and their mechanisms of action have made it necessary to adjust the definitions and criteria for each dementia type. Nevertheless, many genetic markers remain to be studied in order to define whether and how they are involved in a specific NCD type. An example of such limitations is a study of a cohort of 200 VaD patients, 407 late-onset AD patients, and 405 cognitively-healthy control subjects who were genotyped for the C allele of chromosome 9p21.3. Chromosome 9p21.3 was found to be associated with both VaD (95%, *P *< 0.01) and late-onset AD (95%, *P *< 0.01) after adjusting for ApoE ε4 carrier status and other vascular risk factors significantly [67]. Also, a model to predict incident AD did not improve its results after adding CLU and PICALM to the considered variables of age, sex and ApoE, even though these genes have been confirmed to be associated with AD (changes in the Rotterdam Study from 0.847 to 0.849, and from 0.702 to 0.705 in the Cardiovascular Health Study) [29]. By contrast, a study in 207 VaD patients and 207 sex- and age-matched controls found no significant associations between VaD susceptibility and 13 selected AD-associated SNPs: three intergenic variants in chromosomes 9, 15, and 19, and intragenic variants within angiotensin I-converting enzyme (ACE), ApoE (based on haplotypes ε2, ε3, and ε4 of two SNPs), brain derived neurotrophic factor (BDNF; intron 9 and exon 11), death-associated protein kinase 1 (DAPK1), eukaryotic translation initiation factor 2-alpha kinase 2 (EIF2AK2), GRB-associated binding protein 2 (GAB2), and golgi membrane protein 1 (GOLM1; introns 2 and 3) genes [51].

Furthermore, LBD patients have less gray matter atrophy in the medial temporal lobe than AD patients. On the other hand, LBD patients have increased atrophy in subcortical gray matter (i.e., putamen and basal forebrain) and white matter (i.e., dorsal midbrain and pons) than AD patients. Another distinctive feature of LBD is cardiac sympathetic denervation (even in the prodromal state), which is not present in AD. Patients with LBD typically show greater impairment in visuospatial functioning, measures of attention, and executive function compared to AD patients. Additionally, patients are diagnosed with LBD when cognitive impairments or hallucinations occur before, or within a year from the Parkinsonism onset. If Parkinsonism precedes cognitive impairment by more than a year, Parkinson´s disease is diagnosed [55].

Recently discovered genetic factors that are associated with endocrine and mitochondrial dysfunction and may have a role in accelerating disease onset and mechanisms are being studied. Older age, genotypes, and family history are consistent, non-modifiable risk factors for NCD, however, cognitive reserve, cardiovascular function, lifestyle and psychosocial environment are modifiable risk factors and, thus, potential treatment targets [68]. In addition to the main pathological features of AD, deficiencies in S-adenylmethionine (SAM), vitamin B12, and folate are found in these patients. As a result, gene promoter methylation with upregulation of AD-associated genes is triggered by these deficiencies. B vitamin deprivation enhances amyloid-beta deposition in mice [69]. As well, there are environmental risk factors for AD such as cholesterol, diet [70], head trauma [71], posttraumatic stress disorder [72,  73], and reduced levels of exercise [70,  74] that are potentially modifiable. Nevertheless, the highest risk rate for AD is related to the early years of life. Thus, the etiology of dementias, particularly AD, can be explained considering the neuropathological features as well as the environmental factors associated with the disease. Lahiri *et al.* [75] have proposed a Latent Early-Life Associated Regulation (LEARn) multifactorial model, which postulates that epigenetic changes in the expression of specific genes due to the exposure to environmental agents such as diet, toxic substances, and intrinsic factors (e.g., cytokines), produce pathological results only until significantly later in life. Potentially harmful environmental agents may become latent and be present again at maturity or senescence increasing production of Aβ and possibly inducing neurodegeneration and dementia later in life [76]. This model considers not only associated genes but also non-genetic risk factors to explain the AD etiology. Thus, no single factor is considered either necessary or sufficient positing polygenic and multifactorial AD causes [75]. This approach could also be used in order to achieve better understanding of the various NCD etiologies.

In order to delay and prevent disease onset by interventions directed to these modifiable factors, it is necessary to identify dementia before or during its asymptomatic phase, which may be increasingly possible through the assessment of appropriate genetic markers.

This review aims to summarize the definitions, criteria, pathologies, and genetic markers for the most common NCD etiological subtypes (Table **1**). As life expectancy increases worldwide and research on aging-related pathologies advances, a consensus on conceptual and diagnostic criteria acquires more relevance in order to join efforts to develop and implement more accurate and effective treatments.

## CONFLICT OF INTEREST

The authors confirm that this article content has no conflict of interest.

## Figures and Tables

**Fig. (1):**
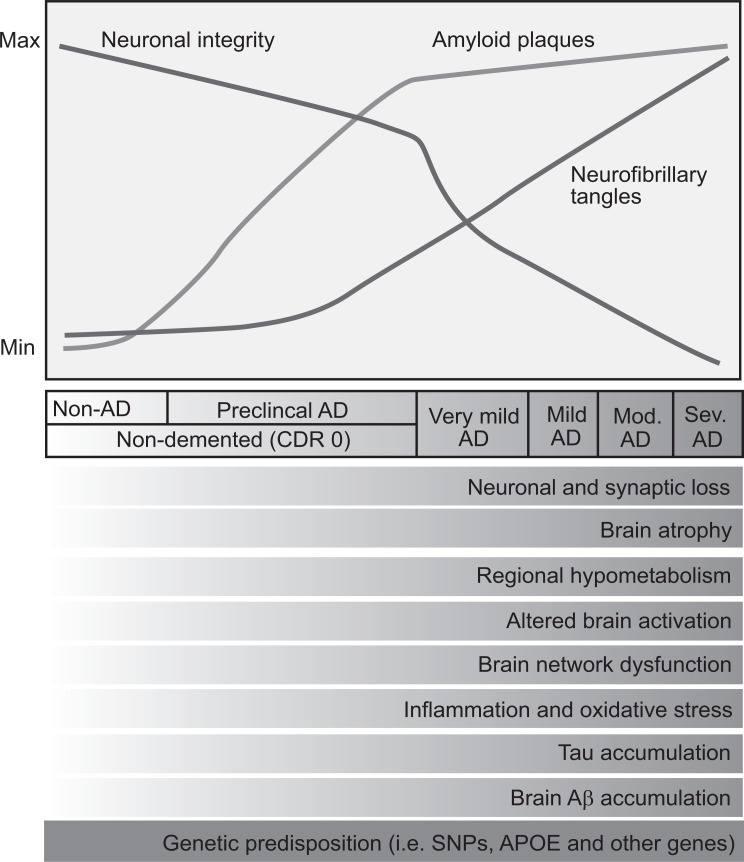
Cognitive, clinical and biomarker changes in AD throughout the evolution of the pathology. Aβ aggregation in the form of amyloid plaques occurs progressively over time in the brain of normal people who later develop AD. The preclinical stage of AD starts approximately 5 years before clear-cut cognitive deterioration. This preclinical stage includes increases in tau accumulation in the neocortex, inflammation and oxidative stress, beginning of neuronal and synaptic loss and brain atrophy, and decline of brain network connections and metabolism. Mild NCD due to AD becomes clinically detectable once neuronal and synaptic dysfunction as well as cell loss have reached some threshold, and amyloid deposition has almost reached its peak. As NCD due to AD progresses, NFT formation as well as neuronal and synaptic dysfunction, inflammation, cell death, and brain atrophy increase. Modified from [12].

**Table 1:** NCD etiological subtypes, their characteristics, pathologies and genetic markers [5, 6].

NCD Etiological Subtype	Characteristics	Neuropathology	Genetic Markers
Alzheimer´s Disease (AD)	Impaired memory, apathy and depression Gradual onset	Cortical amyloid plaques and neurofibrillary tangles	Aβ42 Aβ42 / Aβ40 Tau SNPs (CLU, PICALM, CR1, BIN1, DAB1, CD2AP, TOMM40, EPHA1, CD33, FRMD4A) ApoE ε4
Vascular Disease (VaD)	Similar to AD, but memory is less affected and mood fluctuations more prominent Physical frailty Stepwise onset	Cerebrovascular disease Single infarcts in critical regions, or more diffuse multi-infarct disease	APP Notch3 Cystatin C, GLA, CBS, SYK, TAAR6 Aβ E22Q ApoE ε4
Multiple Etiologies	More than one etiological process (i.e., VaD and AD, or AD and LBD)	Neuropathological features for more than one etiological process	APOE? ??????
Lewy Body Disease (LBD)	Marked fluctuation in cognitive ability Visual hallucinations Parkinsonism (tremor and rigidity)	Cortical Lewy bodies (αSyn)	2q35-q36 GBA1 Annexin A5 ApoE ε4 A-42/A-38 ratio αSyn
Parkinson´s Disease	Gradual cognitive decline following Parkinson´s disease onset Apathy, depressed mood, anxiety REM sleep disorder Excessive daytime sleepiness	Cortical Lewy bodies (αSyn)	αSyn Parkin mutations
Frontotemporal Lobar Degeneration (FTLD)	Personality changes Mood changes Disinhibition Language difficulties	No single pathology -damage limited to frontal and temporal lobes	t-tau, p-tau181 and Aβ42 Aβ42/Aβ40 ratio MAPT, PGRN, C9ORF72, VCP, CHMP2B, TDP-43, FUS, TARDBP Tau
